# Microbial Screening Based on the Mizoroki–Heck Reaction Permits Exploration of Hydroxyhexylitaconic-Acid-Producing Fungi in Soils

**DOI:** 10.3390/microorganisms8050648

**Published:** 2020-04-29

**Authors:** Mei Sano, Ryoki Yada, Yusuke Nomura, Takahiro Kusukawa, Hiroshi Ando, Keiji Matsumoto, Kazuhito Wada, Tomonari Tanaka, Hitomi Ohara, Yuji Aso

**Affiliations:** 1 Department of Biobased Materials Science, Kyoto Institute of Technology, Kyoto 606-8585, Japan; d6861001@edu.kit.ac.jp (M.S.); m7661008@edu.kit.ac.jp (R.Y.); m8661011@edu.kit.ac.jp (Y.N.); t-tanaka@kit.ac.jp (T.T.); ohara@kit.ac.jp (H.O.); 2 Department of Chemistry and Materials Technology, Kyoto Institute of Technology, Kyoto 606-8585, Japan; kusu@kit.ac.jp; 3 Corporate Research & Business Division, Kaneka Corporation, Osaka 530-8288, Japan; Hiroshi.Ando@kaneka.co.jp (H.A.); Keiji.Matsumoto@kaneka.co.jp (K.M.); Kazuhito.Wada@kaneka.co.jp (K.W.)

**Keywords:** itaconic acid, hydroxyhexyl itaconic acid, screening, Mizoroki–Heck reaction

## Abstract

Recently, we developed a unique microbial screening method based on the Mizoroki–Heck reaction for itaconic acid (IA)-producing fungi. This method revealed that 37 out of 240 fungal strains isolated from soils produce vinyl compounds, including IA. In this study, we further characterized these compounds in order to verify that the screening method permits the isolation of fungi that produce other vinyl compounds, excluding IA. HPLC analysis showed that 11 out of 37 isolated strains produced IA, similar to *Aspergillus terreus* S12-1. Surprisingly, the other 8 isolated strains produced two vinyl compounds with HPLC retention times different from that of IA. From these strains, the vinyl compounds of *Aspergillus niger* S17-5 were characterized. Mass spectrometric and NMR analyses showed that they were identical to 8-hydroxyhexylitaconic acid (8-HHIA) and 9-HHIA. This finding showed that 8-HHIA- and 9-HHIA-producing fungi, as well as IA-producing fungi, are ubiquitously found in soils. Neither 8-HHIA nor 9-HHIA showed antibacterial or anti-inflammatory activities. Interestingly, 8-HHIA and 9-HHIA showed cytotoxicity against the human cervical cancer cell line (HeLa) and human diploid cell line (MRC-5), and MRC-5 only, respectively, compared to IA at the same concentration. This study indicates that the screening method could easily discover fungi producing 8-HHIA and 9-HHIA in soils.

## 1. Introduction

Itaconic acid (IA), consisting of a terminal C-C double bond and two carboxyl groups, is a vinyl compound produced by microbes and mammalian macrophages. IA is industrially produced by the fungus *Aspergillus terreus*, and is widely used as a building block for synthetic polymers [1,2,3,4,5], food additives [6], and surfactants [7,8]. *A. terreus* [9,10], *Aspergillus itaconicus* [11], *Helicobasidium mompa* [12], *Ustilago maydis* [13], *Ustilago zeae* [14], *Candida* sp. [15], and *Pseudozyma antarctica* [16] were found to be IA producers in soils, plants, and fermented foods. On the other hand, IA derivatives have also been reported, and they possess a basic structure of alkylitaconic acid or γ-butyrolactone. Butylitaconic acid (produced by *Eupenicillium* sp., *Talaromyces assiutensis*) [17,18], hexylitaconic acid (*Apiospora montagnei, Arthrinium* sp., *Aspergillus niger, Curvularia* sp., *Eupenicillium* sp., *Penicillium* sp.) [17,19,20,21,22,23,24,25,26,27], 8-hydroxyhexylitaconic acid (8-HHIA) (*Aspergillus aculeatus, Penicillium* sp., *T. assiutensis*) [18,20,24], 9-HHIA (*A. niger, Penicillium* sp., *T. assiutensis*) [18,20,21], 8-HHIA-4-methyl ester (*A. aculeatus*) [24], 8-HHIA-1-methyl ester (*A. montagnei*) [22], octylitaconic acid (*Nodulisporium* sp., *Pestalotiopsis theae*) [28,29], ceriporic acids A–H (*Ceriporiopsis subvermispora*) [25,30,31,32,33,34,35], tricladic acids A–C (*Tricladium castaneicola*) [36], deoxysporothric acid (*Nodulisporium* sp.) [28], epideoxysporothric acid (*Nodulisporium* sp.) [28], tensyuic acids A–F (*A. niger*) [37], xylobovide (*Xylaria obovata*) [38], canadensolide (*Penicillium canadense*) [39], sporothriolide (*Nodulisporium* sp.) [40], methylenolactocin (*Penicillium* sp.) [41], and asperitaconic acids A–C (*A. niger*) [42] have been reported as IA derivatives produced by various fungi. It has also been previously reported that IA derivatives show various bioactivities, such as antibacterial, anti-inflammatory, cytotoxic, and plant growth regulatory activities [19,43,44,45,46,47,48,49,50,51,52,53,54,55,56]. Owing to their biological properties, IA and its derivatives are attracting attention as targets of drug discovery.

Typically, IA producers and their derivatives have been isolated using a screening method based on the phenotypes (biological, chemical, and physiological properties) of the products [57]. This conventional method enables the identification of isolated strains as producers of IA and their derivatives only after the structures of the products are characterized, as the producers are unselectively screened by the method. We recently developed a selective screening method for IA producers [58,59]. The screening method is briefly as follows: Soils are plated onto an agar medium and the cultures of the isolated fungal colonies are subsequently subjected to the Mizoroki-Heck reaction with iodobenzene using a palladium catalyst at 80 °C for 1 h. In this reaction, the terminal C–C double bond of IA is specifically labeled with iodobenzene, and iodide is formed as a by-product of the reaction when cultures of IA producers are used. The reaction progression can be confirmed by detection of the formation of iodide molecules with a starch-iodine test. Finally, the labeled IA is detected by HPLC analysis and the isolated fungi are confirmed as IA producers. In addition, an iodide anion is formed as the reaction progresses. This iodide anion can be quickly detected by the starch-iodine test. The iodide anions detected by the starch-iodine test also indicate the concentration of IA present in the culture. This screening strategy is a structure-based screening method based on the structure of the terminal C-C double bond of IA. This method has the advantage of quick and selective screening for IA producers, compared to other conventional methods. 

In our previous study, 240 filamentous colonies were randomly isolated from soils and 37 out of 240 isolated strains were found to produce IA-like vinyl compounds by the structure-based screening method [58]. Of these 37 isolated strains, the vinyl compound produced by *A. terreus* S12-1 was characterized after labeling with iodobenzene using the Mizoroki–Heck reaction strategy. Mass spectrometric (MS) analysis showed that the monoisotopic mass and molecular formula of the vinyl compound produced were 206.06 [M-H]^−^ and C_11_H_10_O_4_, respectively, resulting in the compound being identified as IA. The Mizoroki–Heck reaction can also label acrylic acid, indicating that the developed screening method can also detect vinyl compounds other than IA [60]. However, there is no report on the screening of microbes producing vinyl compounds other than IA using this screening method.

The present study was carried out to analyze vinyl compounds produced by these 37 strains, including *A. terreus* S12-1. In addition, we also verified that the developed screening method enables the screening of microbes producing vinyl compounds other than IA. As a result, 11 new strains along with S12-1 were identified as IA producers, and 8 strains were microbes that produce vinyl compounds other than IA. The S17-5 strain was one of eight strains producing two vinyl compounds other than IA. The vinyl compounds produced by S17-5 were identified by nuclear magnetic resonance (NMR) and liquid chromatography-mass spectrometry (LC-MS), and their antibacterial, anti-inflammatory, and cytotoxic properties were evaluated. Based on the results, *A. niger* S17-5 produces compounds 8-HHIA and 9-HHIA. Thus, there are almost an equal number of IA producers and 8-HHIA and 9-HHIA producers in soil. In addition, 8-HHIA and 9-HHIA showed cytotoxicity against human cervical cancer cell line (HeLa) and human diploid cell line (MRC-5), and MRC-5 only, respectively. These results indicate that the screening method based on the Mizoroki–Heck reaction enables the screening of microbes producing vinyl compounds other than IA.

## 2. Materials and Methods

### 2.1. Isolation of Fungi from Soils

Forty-eight soil samples from several places in Japan were separately mixed with sterilized water and then plated on potato-dextrose broth (Becton, Dickinson and Company, Sparks, MD, USA) agar plates supplemented with 25 µg/mL chloramphenicol. The soil fungus S17-5 strain was isolated from Kamiyagawa-cho, Sakyo-ku, Kyoto, Japan. After incubation for 7 days at 30 °C, five colonies per soil sample were isolated and separately cultivated in 0.7 mL GM1 liquid medium (20 g/L glycerol, 0.154 g/L MgSO_4_ 7H_2_O, 0.19 mg/L FeCl_2_ 4H_2_O, 0.46 g/L NH_4_NO_3_, 15.4 mg/L KH_2_PO_4_, 96 mg/L CaCl_2_, 1.2 mg/L ZnSO_4_ 7H_2_O, and 2.3 mg/L CuSO_4_ 5H_2_O) in a 96-well deep plate (vol: 1.1 mL; Ina Optika Co., Ltd., Osaka, Japan) covered with a plate seal (Thermo Fisher Scientific, Waltham, MA, USA) using a plate shaker (DWMax M BR-034P, TAITEC, Saitama, Japan) at 30 °C and 1600 rpm for 7 days.

After cultivation, the cultures were screened based on the Mizoroki–Heck reaction [45]. For the labeling reaction, aliquots of each culture (10 μL), along with 180 mM iodobenzene (IB) in dimethyl sulfoxide (DMSO) (10 μL), 375 mM K_2_CO_3_ in water (4 μL), and 4.5 mM Pd(OAc)_2_ in DMSO (2 μL) were added to a 96-well PCR plate (volume 0.1 mL; Ina Optika, Osaka, Japan) and heated at 80 °C for 1 h. Concentrated HCl (1.75 μL) was added to the mixture to adjust the pH of the mixture to 1.0. After the labeling reaction, 50 µL of 5% soluble starch and 5% NaNO_2_ were added to the reaction mixture and cooled at −20 °C for 10 min. The reaction transition was monitored using a starch-iodine test in a 96-well microplate. Ten microliters of the reaction mixture showing coloration were injected and monitored using high-performance liquid chromatography (HPLC) (LaChrom Elite, Hitachi High-Tech Corporation, Tokyo, Japan) equipped with a COSMOSIL 5C18-AR-II column (Nacalai Tesque, Kyoto, Japan). A linear gradient elution from 0% acetonitrile containing 0.01% TFA to 100% acetonitrile containing 0.01% TFA for 20 min was performed. The flow rate was 1 mL/min, and the absorbance of the eluate was monitored at 210 nm.

### 2.2. Identification of S17-5 Strain

The isolated S17-5 strain was identified based on the ribosomal RNA (rRNA) gene sequence. Partial regions of the 26S rRNA gene and rRNA intergenic spacer were amplified and sequenced with the genomic DNA extracted from S17-5 as a template using two sets of primers: NL1 (5′- GCATATCAATAAGCGGAGGAAAAG-3′) and NL4 (5′-GGTCCGTGTTTCAAGACGG-3′), and ITS1 (5′-TCCGTAGGTGAACCTGCGG-3′) and ITS4 (5′-TCCTCCGCTTATTGATATGC-3′). DNA sequencing was performed by Macrogen Inc. (Kyoto, Japan).

### 2.3. Purification of Vinyl Compounds Produced by S17-5 Strain Culture

The isolated fungal strain was cultivated in 60 mL GM2 liquid medium (130 g/L glucose, 1 g/L MgSO_4_ 7H_2_O, 1.25 mg/L FeCl_2_ 4H_2_O, 3 g/L NH_4_NO_3_, 0.1 g/L KH_2_PO_4_, 625 mg/L CaCl_2_, 8 mg/L ZnSO_4_ 7H_2_O, and 15 mg/L CuSO_4_ 5H_2_O) in a 500 mL baffled shake flask at 30 °C and 120 rpm for 10 days. After cultivation, the culture was filtered through a filter paper (No. 5A, Advantec, Tokyo, Japan) to obtain the culture supernatant. The supernatant was further filtered through a PES syringe filter (pore size 0.45 μm). The 8-HHIA and 9-HHIA in the culture supernatant were purified using a LaChrom Elite HPLC equipped with a preparative HPLC Inertsil ODS 10 µm column (GL sciences, Tokyo, Japan). The HPLC steps used for elution were as follows: An initial equilibration and injection step at 10% acetonitrile containing 0.01% TFA for 15 min was performed followed by an elution step using 15% acetonitrile containing 0.01% TFA for 15 min. The flow rate was kept at 5 mL/min, and the absorbance of the eluate was monitored at 210 nm. The eluates corresponding to 8-HHIA and 9-HHIA were obtained and freeze-dried, resulting in the collection of 20 mg of 8-HHIA and 9-HHIA. Using purified 8-HHIA and 9-HHIA as standards, the concentrations of 8-HHIA and 9-HHIA in the culture supernatant were quantified by HPLC analysis. The purified 8-HHIA and 9-HHIA were dissolved in methanol and MeOD containing 0.05% tetramethylsilane for LC-MS and NMR analyses, respectively.

### 2.4. Structural Characterization of the Two Vinyl Compounds Produced by S17-5

LC-MS was performed using Prominence HPLC (Shimadzu, Kyoto, Japan) equipped with a Cadenza CD-C18 column (Imtakt, Kyoto, Japan) and an ESI-MS detector micrOTOF-Q II (Bruker Daltonics, Billerica, MA, USA). After the labeling reaction, 10 μL of the mixture was injected and monitored. A linear elution gradient from 0% acetonitrile containing 0.1% formic acid to 100% acetonitrile containing 0.1% formic acid for 20 min was performed. The flow rate was maintained at 0.2 mL/min, and the absorbance of the eluate was monitored at 210 nm. MS analysis was performed in the negative ion mode.

The structural assignment of 8-HHIA and 9-HHIA was performed using a 500 MHz Bruker AVANCE III NMR system (Bruker Biospin, Billerica, MA, USA) with ^1^H-^13^C heteronuclear multiple quantum correlation (HMQC), distortionless enhancement by polarization transfer (DEPT), heteronuclear multiple bond correlation (HMBC), and two-dimensional correlated spectroscopy (COSY) NMR experiments.

### 2.5. Antibacterial Test

The minimum inhibitory concentrations (MICs) of 8-HHIA and 9-HHIA were tested against six pathogenic bacteria: *Escherichia coli* NBRC 3972, *Pseudomonas aeruginosa* NBRC 12689, methicillin-resistant *Staphylococcus aureus* IID 1677, *Salmonella enteritidis* NBRC 3313, *Vibrio parahaemolyticus* NBRC 12711, and *Klebsiella pneumoniae* NBRC 13277. The 8-HHIA and 9-HHIA solutions (2.5 mM) were serially diluted with distilled water and added to BBL Mueller Hinton Broth (Becton, Dickinson and Company, Cockeysville, MD, USA). After inoculation of the indicator strains, the cultures were incubated at 35 °C for 1 day. This test was performed at Kyoto Biken Laboratories, Inc. (Kyoto, Japan).

### 2.6. Anti-Inflammatory Test

The murine macrophage cell line RAW264 (5 × 10^4^ cells) was seeded in 1 mL of DMEM (Wako Pure Chemical Industries, Osaka, Japan) containing 10% fetal bovine serum (FBS) (Biosera, Ringmer, UK) and 1% non-essential amino acids (NEAA; Wako Pure Chemical Industries Ltd., Osaka, Japan) in a 24-well plate (TrueLine, Nippon Genetics, Toyama, Japan) under 5% CO_2_ at 37 °C. After 24 h of incubation, DMSO, IA, 8-HHIA, and 9-HHIA (0.01 and 0.1 mM) compounds were separately added to the cultures, followed by further incubation. After 2 h of incubation, 10 µL of 0.1 mg/mL lipopolysaccharide (LPS) (Sigma-Aldrich, St Louis, MO, USA) in phosphate-buffered saline solution (without Ca^+^ and Mg^+^) was added to each culture. After 24 h of incubation, the cultures were monitored at 450 nm using an iMark spectrophotometric plate reader (Bio-Rad, Hercules, CA, USA). The titers of IL-1β and IL-6 produced in the culture were quantified using ELISA kits (ProteinTech Group, Chicago, IL, USA), according to the manufacturer’s instructions. Cytokine production was defined as 100% when DMSO was added to the cultures after induction with LPS. This assay was performed in triplicates.

### 2.7. Cytotoxicity Test

The human cervical cancer cell line HeLa and normal embryonic lung fibroblast cell line MRC-5 (4 × 10^4^ cells) were seeded in 100 µL of medium (DMEM with 10% FBS and 1% NEAA) in 96-well plates (TrueLine) under 5% CO_2_ at 37 °C. After 18 h of incubation, DMSO, IA, 8-HHIA, and 9-HHIA (0.01 and 0.1 mM) were separately added to the cultures, followed by further incubation. After 24 h of incubation, the cell viability was measured based on a colorimetric method using a Cell Counting Kit-8 (CCK-8) assay kit (Dojindo Corp., Kumamoto, Japan). CCK-8 solution (10 µL) was added to the cultures, followed by incubation for 30 min. The absorbance of the samples was read at 450 nm using a spectrophotometric plate reader (iMark, Bio-Rad). Cell viability was defined at 100% when DMSO was added to the cultures. This assay was performed in hexaplicate.

## 3. Results and Discussion

### 3.1. Characterization of Vinyl Compounds Produced by 37 Fungi Isolated from Soils

**Figure 1:**
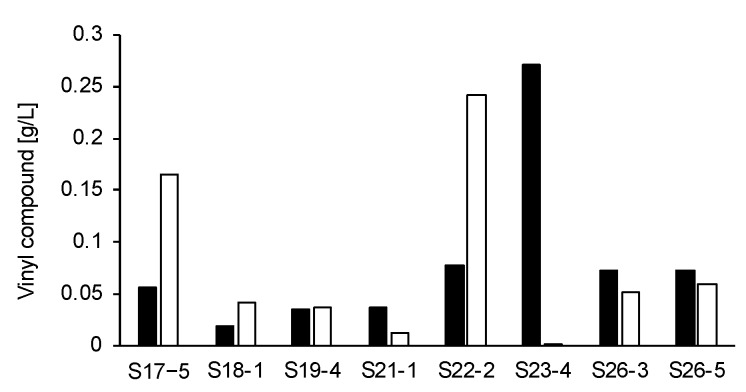
Production of vinyl compounds A and B by eight fungal strains. The concentrations of compounds A and B are represented by open bars and solid bars, respectively. Eight strains were cultivated in 0.7 mL GM1 liquid medium for 7 days at 30 °C and 1600 rpm.

**Figure 2:**
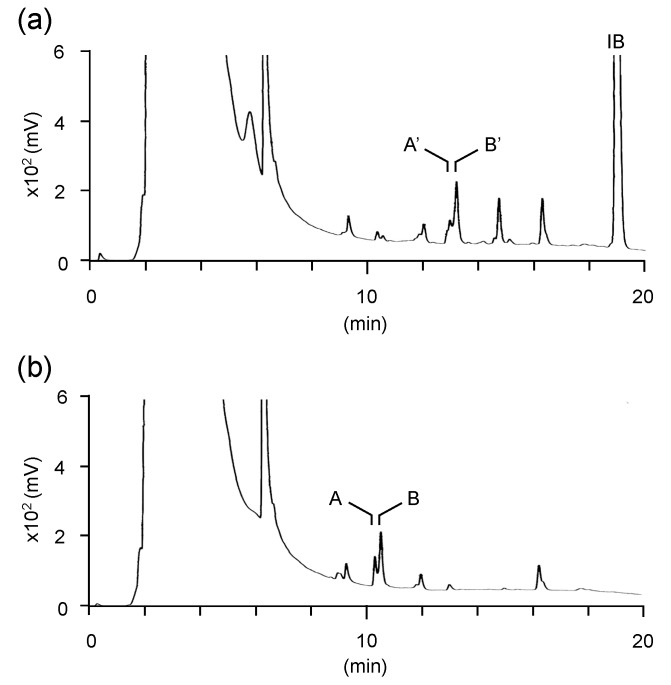
HPLC analysis of the culture supernatant of S17-5. (**a**) The culture supernatant after 7 days of cultivation was labeled based on the Mizoroki–Heck reaction. Peaks A’ and B’ correspond to the IB-labeled compounds A and B, respectively. Peak IB corresponds to residual IB after the Mizoroki–Heck reaction. (**b**) The culture supernatant after 7 days of cultivation was analyzed without the labeling reaction. Peaks A and B correspond to compounds A and B, respectively.

In the current study, vinyl compounds produced by 37 different strains of fungi, cultivated in 0.7 mL GM1 liquid medium (20 g/L glycerol, 0.154 g/L MgSO_4_ 7H_2_O, 0.19 mg/L FeCl_2_ 4H_2_O, 0.46 g/L NH_4_NO_3_, 15.4 mg/L KH_2_PO_4_, 96 mg/L CaCl_2_, 1.2 mg/L ZnSO_4_ 7H_2_O, and 2.3 mg/L CuSO_4_ 5H_2_O) for 7 days at 30 °C and 1600 rpm, were identified by HPLC analysis. As a result, we identified that 11 new strains, along with S12-1, were IA producers and 8 strains were microbes producing two vinyl compounds other than IA. IA was observed at a retention time (RT) of 7.5 min on HPLC chromatogram, and the IB-labeled vinyl compound was observed at an RT of 12.5 min on HPLC chromatogram [58]. On the other hand, two vinyl compounds produced by these eight strains were observed consistently at an RT of 10.3 and 10.5 min, and the corresponding IB-labeled vinyl compounds were observed at an RT of 12.9 and 13.2 min. Hereafter, the vinyl compounds produced by 8 strains are referred to as compounds A and B. Among the eight strains, S17-5, S22-2, and S23-4 produced more these vinyl compounds than the other strains. The concentrations of compounds A and B in the culture supernatant of S17-5, S22-2, and S23-4 after 7 days of cultivation were 0.06 g/L and 0.17 g/L, 0.08 g/L and 0.24 g/L, and 0.27 g/L and 0.002 g/L, respectively (Figure 1). The S17-5 strain stably produced two vinyl compounds A and B. Thus, the S17-5 strain was used as a representative producer of the two vinyl compounds. The vinyl compounds A and B produced by strain S17-5 were observed at RTs of 10.3 and 10.5 min, respectively, on the HPLC chromatogram (Figure 2). Compounds A and B were classified, and their bioactivities were evaluated. An analysis of 26S rRNA genes resulted in the identification of the S17-5 strain as *A. niger*. The other 18 out of 37 strains analyzed produced no vinyl compounds. The reason why these 18 strains were screened as vinyl compound producers is because the Ullmann reaction progressed instead of the Mizoroki–Heck reaction during the labeling process. The Ullmann reaction is a synthesis reaction of biphenyl, wherein two aryl halides react in the presence of a palladium catalyst, a base and a reductant such as ethanol [61]. Fungal metabolites including ethanol could act as reductants. The progression of the Ullmann reaction resulted in a false recognition of the progression of the Mizoroki–Heck reaction. These results indicate that fungi isolated from soil were briefly categorized into three types: IA producers, producers of vinyl compounds other than IA, and microbes not producing vinyl compounds. 

### 3.2. Structural Characterization of Vinyl Compounds A and B Produced by S17-5

**Figure 3:**
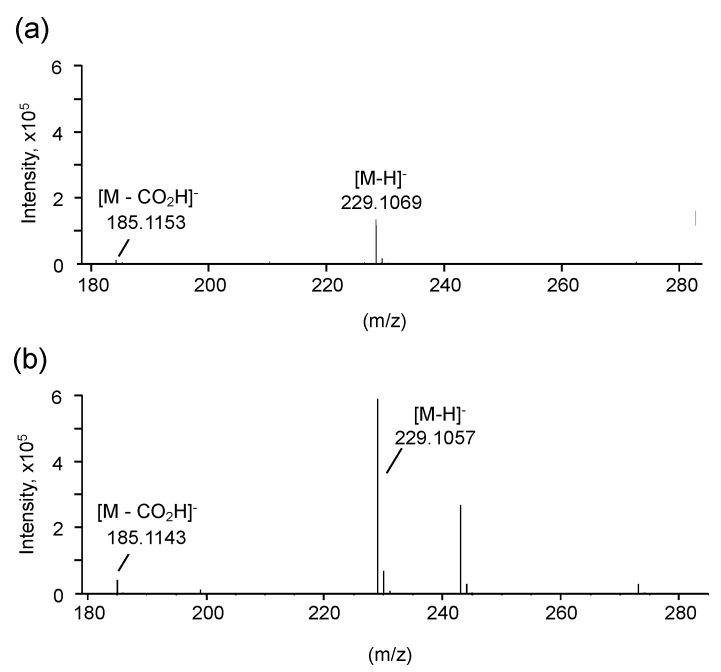
Mass analyses of compounds A (**a**) and B (**b**). Mass analyses were performed in the negative ion mode.

Mass analysis showed that the monoisotopic mass and molecular formula of compound A were 229.1069 [M-H]^−^ (calculated for C_11_H_17_O_5_, 229.2497) and C_11_H_18_O_5_, respectively (Figure 3a). The presence of a carbonyl group was confirmed, since the adduct ion corresponding to decarboxylated anions was observed (185.1153 [M-H]^−^). The monoisotopic mass and molecular formula of compound B were 229.1057 [M-H]^−^ (calculated for C_11_H_17_O_5_, 229.2497) and C_11_H_18_O_5_, respectively (Figure 3b). Additionally, adduct ions corresponding to decarboxylated anions were observed (185.1143 [M-H]^−^). Decarboxylation indicated that compounds A and B were carboxylic acids.

**Figure 4:**
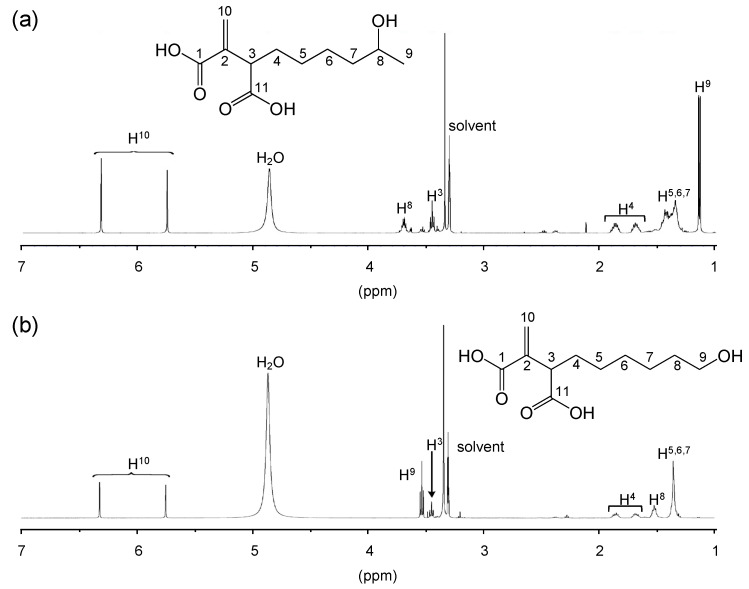
The ^1^H NMR spectra of compounds A (**a**) and B (**b**).

The chemical structures of compounds A and B were identified by ^1^H and ^13^C NMR analyses. The ^1^H NMR spectroscopic data of compounds A and B are shown in Figure 4 and Table S1. According to the molecular formula and MS data, compound A has one hydroxyl group and two carboxyl groups. The ^13^C NMR spectra of compound A showed resonances at δ_C_ 169.6 and 177.1. This supports the presence of carbonyl groups in compound A. The ^13^C-^13^C couplings of C-4 and C-5 signals (δ_C_ 32.2 (d, 5.0 Hz) and 28.7 (d, 5.0)) indicate the presence of a carbonyl group adjacent to C-4 and C-5. The ^1^H NMR spectrum of compound A showed signals of exo-methylene protons at δ_H_ 5.75 and 6.32 (H^10^); two sp^3^ methines at δ_H_ 3.45 and 3.70 (H^3^ and H^8^); four methylenes at δ_H_ 1.40, 1.69, and 1.87 (H^4^-H^7^); and a methyl group at δ_H_ 1.14 (H^9^) (Figure 4a). Based on these data, compound A was identified as 8-HHIA.

According to the molecular formula and MS data described above, compound B has one hydroxyl group and two carboxyl groups. The ^13^C NMR spectra of compound B showed resonances at δ_C_ 169.6 and 177.2. This supports the presence of carbonyl groups in compound B. The ^1^H NMR spectrum of compound B showed signals for exo-methylene protons at δ_H_ 5.75 and 6.32 (H^10^), two sp^3^ methines at δ_H_ 3.45 (H^3^), and six methylenes at δ_H_ 1.36, 1.52, 1.68, 1.86, and 3.53 (H^4^-H^9^). Based on these data, compound B was identified as 9-HHIA (Figure 4b).

In addition, ^1^H and ^13^C NMR spectra of 8-HHIA and 9-HHIA agreed with the previously reported NMR spectra of these compounds [20,21,24].

### 3.3. Bioactive Characterization of 8-HHIA and 9-HHIA

The antibacterial and anti-inflammatory activities and cytotoxicity of purified 8-HHIA and 9-HHIA were tested. Based on the results obtained, all MICs against six pathogenic bacteria, *E. coli* NBRC 3972, *P. aeruginosa* NBRC 12689, methicillin-resistant *S. aureus* IID 1677, *S. enteritidis* NBRC 3313, *V. parahaemolyticus* NBRC 12711, and *K. pneumoniae* NBRC 13277 were observed at more than 2.5 mM, which was the maximum concentration used in this test. These results indicate that 8-HHIA and 9-HHIA showed no antibacterial activity against these bacteria.

The anti-inflammatory activities of 8-HHIA and 9-HHIA against murine macrophage cells, RAW264, were characterized using ELISA. Compared to IA, 8-HHIA and 9-HHIA showed no anti-inflammatory activities (Figure S1). On the other hand, IA indicated anti-inflammatory activity caused by inhibition of the release of IL-1β in murine macrophage RAW264.7 cells [51,52]. In this study, a similar result was obtained. The hydroxyhexyl group is suggested to be involved in the loss of anti-inflammatory activity as 8-HHIA and 9-HHIA indicated no anti-inflammatory effects compared to IA. Li et al. reported that the anti-inflammatory activities of methyl ester compounds 8-HHIA and 9-HHIA inhibited the release of IL-1β in murine macrophage RAW264.7 cells [20]. In addition, their study also suggested that the ester forms of 8-HHIA and 9-HHIA, rather than the free-acid forms, exhibited anti-inflammatory activities due to the increase in cell permeability by esterification. In their report, 8-HHIA and 9-HHIA decreased anti-inflammatory activities by loss of cell permeability.

**Figure 5:**
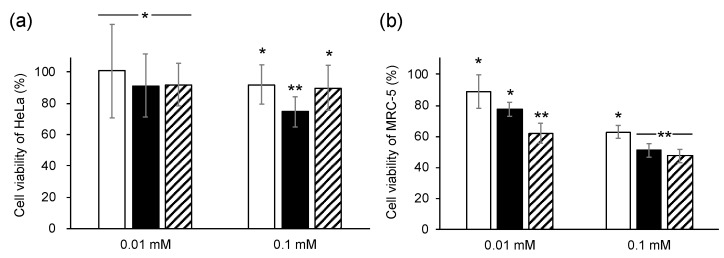
Cytotoxicity of 8-HHIA and 9-HHIA against HeLa and MRC-5 cells. The cell viabilities of HeLa (**a**) and MRC-5 (**b**) cells were determined after the addition of IA (open bars), 8-HHIA (solid bars), and 9-HHIA (hatched bars) in the cultures. Each assay was performed in hexaplicate and the average is represented with error bars indicating standard deviations. Cell viability was defined as 100% when DMSO was added as negative control to the cultures. * *p* > 0.05 and ** *p* < 0.01 vs. treatment with only DMSO.

The cytotoxicity of 8-HHIA and 9-HHIA against a human cervical cancer cell line (HeLa) and a human diploid cell line (MRC-5) was characterized. The 8-HHIA and 9-HHIA compounds exhibited higher cytotoxicity against both HeLa and MRC-5 cell lines and MRC-5 only, respectively, compared to the addition of IA at the same concentrations (Figure 5). IA derivatives, tricladic acids A and C, have 10- and 11-hydroxyoctenyl groups, respectively, indicating that these structures are similar to 8-HHIA and 9-HHIA. Tricladic acid A showed a stronger cytotoxicity against B16 murine melanotic melanoma cells when compared to tricladic acid C [36]. These results suggest that the cytotoxicity of IA derivatives is affected by the positions of the hydroxyl group on the alkyl chain, resulting in a higher cytotoxicity of 8-HHIA when compared to 9-HHIA. 

## 4. Conclusions

Eight strains (3.7%) of IA producers and 11 strains (4.6%) of 8-HHIA and 9-HHIA producers were screened from 240 randomly isolated strains from soil. This indicated that the screening method based on the Mizoroki-Heck reaction enables the screening for 8-HHIA and 9-HHIA producers, which is similar to the screening for IA producers. The 8-HHIA and 9-HHIA compounds indicated no antibacterial or anti-inflammatory activities. However, 8-HHIA showed cytotoxicity against both HeLa and MRC-5 cells, while 9-HHIA showed cytotoxicity to MRC-5 only. These results indicate that the two compounds exhibit different bioactivities compared to IA. We conclude that producers of IA derivatives could be screened from soils using this screening method. This method would provide new drug discovery opportunities.

## Supplementary Materials

The following are available online at https://www.mdpi.com/2076-2607/8/5/648/s1, Figure S1: Anti-inflammatory activities of 8-HHIA and 9-HHIA against RAW264 cells, Table S1: ^1^H and ^13^C NMR spectroscopic data of 8-HHIA and 9-HHIA.
