# Correction: Sano, M., et al. Microbial Screening Based on the Mizoroki–Heck Reaction Permits Exploration of Hydroxyhexylitaconic-Acid-Producing Fungi in Soils. Microorganisms 2020, *8*, 648

**DOI:** 10.3390/microorganisms8060913

**Published:** 2020-06-16

**Authors:** Mei Sano, Ryoki Yada, Yusuke Nomura, Takahiro Kusukawa, Hiroshi Ando, Keiji Matsumoto, Kazuhito Wada, Tomonari Tanaka, Hitomi Ohara, Yuji Aso

**Affiliations:** 1 Department of Biobased Materials Science, Kyoto Institute of Technology, Kyoto 606-8585, Japan; d6861001@edu.kit.ac.jp (M.S.); m7661008@edu.kit.ac.jp (R.Y.); m8661011@edu.kit.ac.jp (Y.N.); t-tanaka@kit.ac.jp (T.T.); ohara@kit.ac.jp (H.O.); 2 Department of Chemistry and Materials Technology, Kyoto Institute of Technology, Kyoto 606-8585, Japan; kusu@kit.ac.jp; 3 Corporate Research & Business Division, Kaneka Corporation, Osaka 530-8288, Japan; Hiroshi.Ando@kaneka.co.jp (H.A.); Keiji.Matsumoto@kaneka.co.jp (K.M.); Kazuhito.Wada@kaneka.co.jp (K.W.)

The authors wish to make the following correction to this paper [1]: the compound names ‘8-hydroxyhexylitaxconic acid’ and ‘9-hydroxyhexylitaconic acid’ should be ‘9-hydroxyhexylitaxconic acid’ and ‘10-hydroxyhexylitaconic acid’, respectively, throughout the manuscript. For this correction, the text in the graphical abstract and the carbon numbering in Figure 4 should be changed. The authors would like to replace the original graphical abstract and Figure 4: 
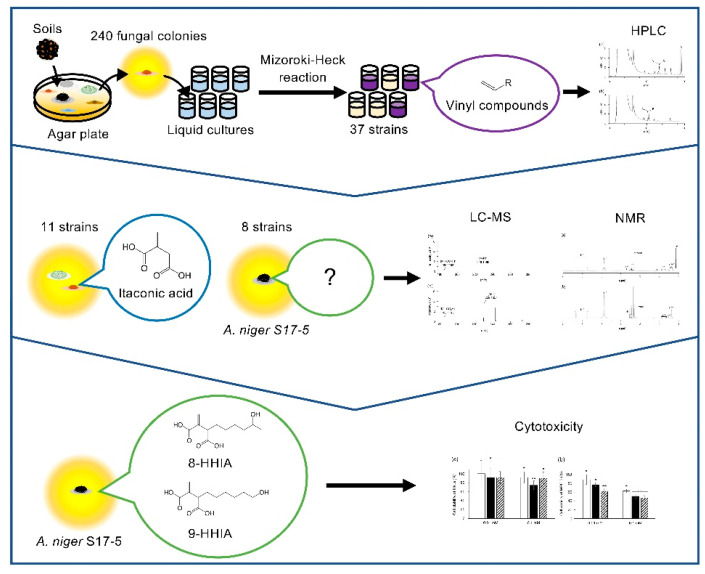
 
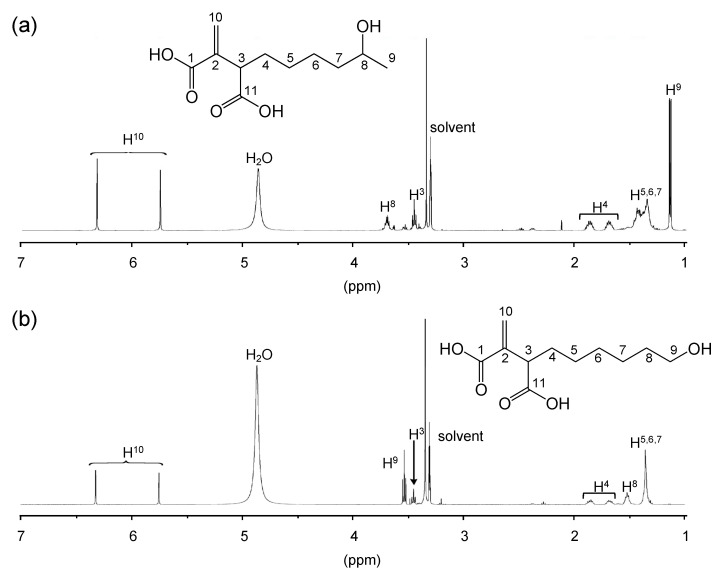
 with new updated graphical abstract and Figure 4, respectively:

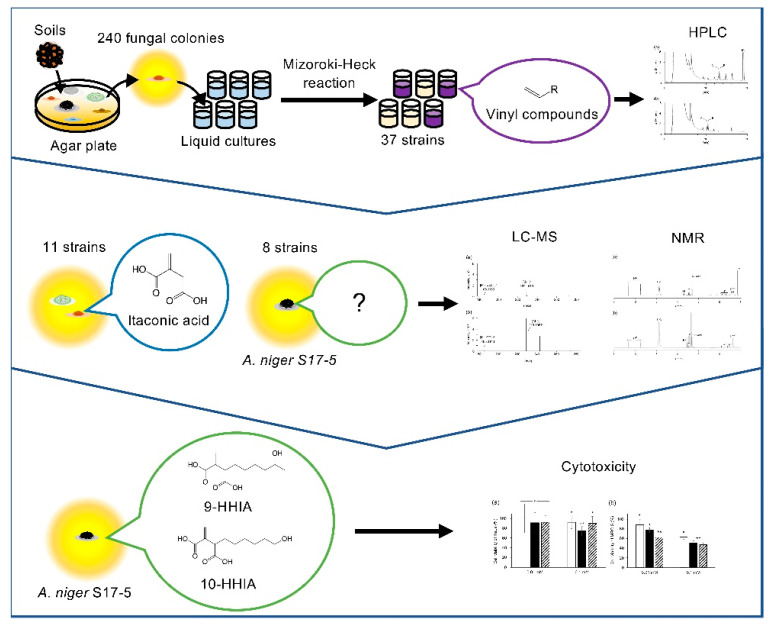

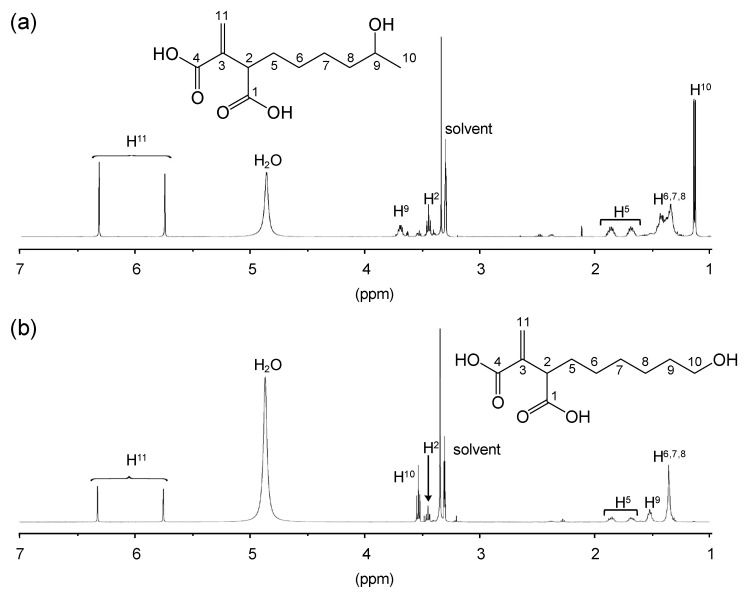





The manuscript will be updated, and the original will remain online on the article webpage, with a reference to this correction. The authors would like to apologize for any inconvenience caused to the readers by this change.
