# Supplementary material for: Microbial Screening Based on the Mizoroki–Heck Reaction Permits Exploration of Hydroxyhexylitaconic-Acid-Producing Fungi in Soils
Source: Microorganisms. 2020 Apr 29;8(5):648. doi: 10.3390/microorganisms8050648 (PMC7284703; doi:10.3390/microorganisms8050648)
Supplement: Supplementary file 1 [file microorganisms-08-00648-s001.pdf]

Table S1. ^{1}H and ^{13}C NMR spectroscopic data of 8-HHIA and 9-HHIA

Position	8-HHIA		9-HHIA	
	δ_H (J in Hz)	δ_C	δ_H (J in Hz)	δ_C
1	–	169.6	–	169.6
2	–	141.0	–	141.0
3	3.45 (t, 7.4)	48.1	3.45 (t, 7.4)	48.1
4	1.69 (m); 1.87 (m)	32.2 (d, 5.0)	1.68 (m); 1.86 (m)	32.2
5	1.40 (m)	28.7 (d, 5.0)	1.36 (m)	28.7
6	1.40 (m)	26.6	1.36 (m)	30.3
7	1.40 (m)	40.0	1.36 (m)	26.7
8	3.70	68.5	1.52 (m)	33.5
9	1.14 (d, 6.2)	23.5	3.52 (t, 6.6)	62.9
10	5.75; 6.32	126.9	5.75; 6.32 (d, 0.54)	126.9
11	–	177.1	–	177.2

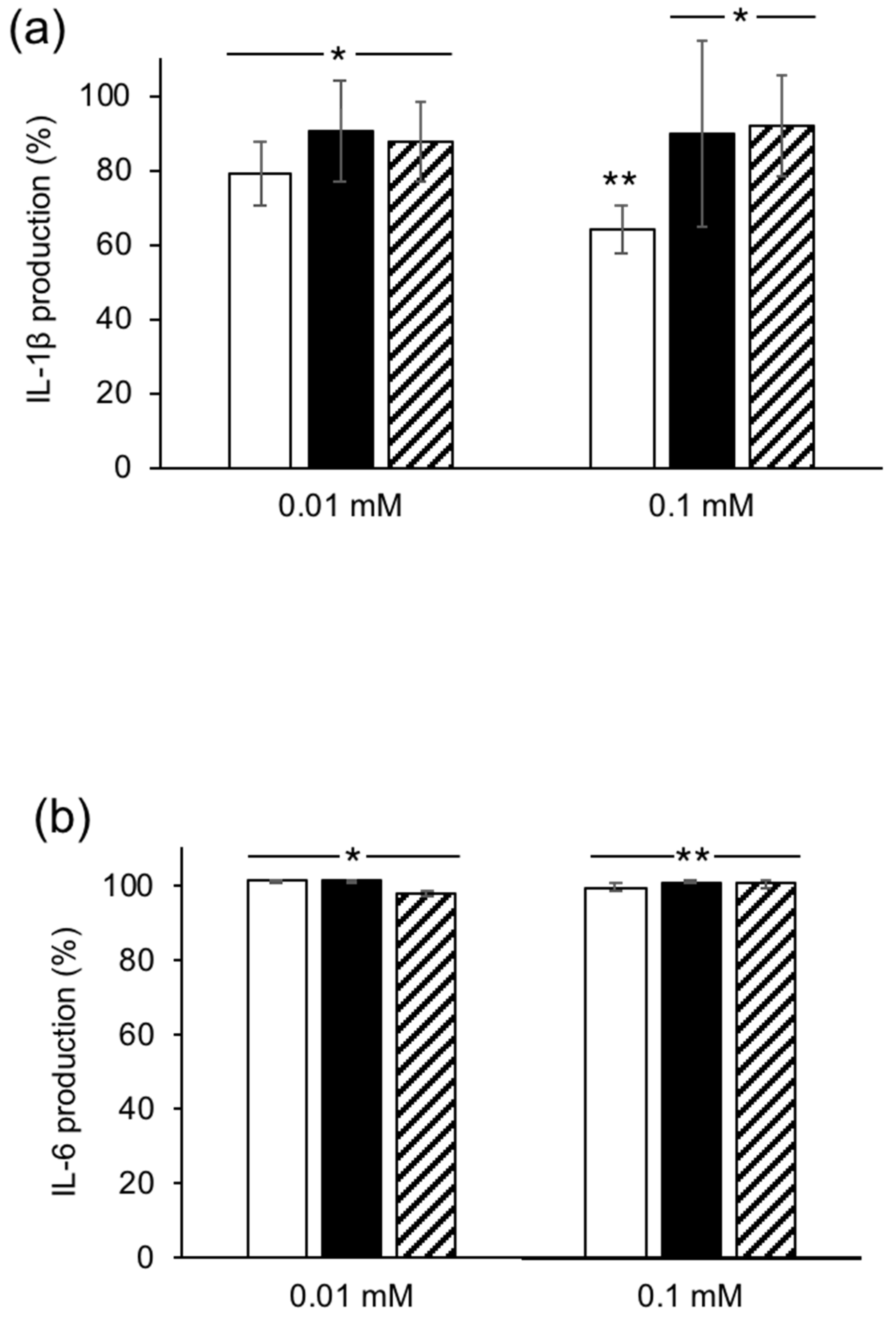

Figure 1. Anti-inflammatory activities of 8-HHIA and 9-HHIA against RAW264 cells. The production levels of cytokines IL-1β (a) and IL-6 (b) in RAW264 cells after induction with LPS were determined in the presence of IA (open bars), 8-HHIA (solid bars), and 9-HHIA (hatched bars). This assay was performed in triplicates and the average value is represented with error bars indicating standard deviations. Cytokine production was defined as 100% when DMSO was added to the cultures

after induction with LPS. *$P > 0.05$ and **$P < 0.01$ vs. treatment with only DMSO after induction with LPS.
